# Mode of Conducting a Pathological Analysis of the Blood

**Published:** 1846-09

**Authors:** 


*Mode of conducting a pathological Analysis of the Blood.*—In the present number of our Journal will be found an interesting article, by Dr. Buck of this city, on the blood in several cases of disease. Deeming it likely that some of our enterprising young friends may be stimulated, by Dr. B's. praiseworthy example, to institute similar inquiries, we transfer from the British and Foreign Medical Review directions for conducting such experiments.

1. A glass containing exactly 1000 grains of water is filled with blood directly from the vein: a glass stopper fitting hermetically, is then inserted (care being taken to remove all bubbles), and the specific gravity of the blood ascertained. By continually shaking it, the blood is kept fluid; it is then poured into a porcelain vessel; and evaporated, care being taken to wash the glass out with distilled water, and add the latter to the other. The quantity of solid residue is reduced to the proportions in 1000 grains of blood. 2. A glass two and a half ounce measure is filled with the blood immediately after the preceding, covered and set aside for eighteen hours. The clot is then carefully separated from the serum, and the weight of both ascertained. 3. The clot is now to be pressed through a linen cloth four inches square, and first the fibrine, then the fluid cruor collected. Each is thoroughly washed, freed from moisture, and weighed; they are then carefully dried, and again weighed. The results are to be reduced to the proportion in 1000 grains of blood. 4. The serum and clot are to be placed in a glass containing 360 grains of distilled water, and their specific gravity noted; then 250 grains of each, at the same temperature, are to be dried so long as they lose weight; the weight of the residuum is then to be reduced to the proportion in 1000 grains. There is loss of weight by pressure through the linen, but as this will happen in the same proportion in all instances, the general results are correct.

Dr. Zimmerman observes that this method will be more suitable to the practical physician than Andral and Gavarett's, because of its greater simplicity and easier performance, while the results are quite as correct as by the latter method.

